# “We started to care for our colleagues”: A qualitative study of statements by physicians and nurses from a COVID-19 ICU of a public university hospital in the Southeast region of Brazil

**DOI:** 10.1371/journal.pmen.0000248

**Published:** 2025-02-11

**Authors:** Felipe Santos da Silva, Luciane Miranda Guerra, Lucas Serra Valladão, Carla Fabiana Casagrande, Jasmine de Matos Cavalcante, Paula Elias Ortolan, Diego Romaioli, Milena Rossi Suedt, Egberto Ribeiro Turato

**Affiliations:** 1 Member of the Laboratory of Clinical-Qualitative Research, School of Medical Sciences, Universidade Estadual de Campinas, São Paulo, Brazil; 2 Department of Health Sciences and Children’s Dentistry, Piracicaba Dental School–Universidade Estadual de Campinas–FOP/UNICAMP, Piracicaba, São Paulo, Brazil; 3 Graduation in Medicine at School of Medical Sciences, Universidade Estadual de Campinas, São Paulo, Brazil; 4 Researcher at the Department of Philosophy, Sociology, Education and Applied Psychology in University of Padua, Padua, Italy; 5 Department of Psychiatry, School of Medical Sciences, Universidade Estadual de Campinas—FCM/UNICAMP, Campinas, São Paulo, Brazil; PLOS: Public Library of Science, UNITED KINGDOM OF GREAT BRITAIN AND NORTHERN IRELAND

## Abstract

The COVID-19 pandemic triggered countless intense emotional experiences. The challenges posed by the unpredictability of this macro-phenomenon led to a “mass illness”, with impacts on mental health, particularly among healthcare providers, who worked directly with hospitalized patients, tirelessly seeking their recovery. This study aimed to interpret the symbolic meanings of statements expressed by physicians and nurses of a COVID-19 intensive care unit to assess their care experiences. This was a humanistic study using the Clinical-Qualitative method (CQM). Data were interpreted using Clinical-Qualitative Content Analysis. Six interviews were performed, which produced the following categories: Category 1 –Psychic time and rudimentary symbolizations on personal experience during the pandemic; Category 2 –Denial as a psychological defense or a psychosocial adaptation mechanism? Category 3 –Tension and family support: triggers of ambivalent experiences; and 4: The feeling of insecurity: from technique to affective dimension. Our findings indicate rudimentary symbolization of experiences in an intense context such as the COVID-19 pandemic. The interviewees presented discursive speech, with poor symbolic expression, as the process for psychic and cultural representation requires time, unlike the chronology of facts that respects the order in which they happen.

## Introduction

On January 23, 2020, the World Health Organization (WHO) set a meeting with the Emergency Committee and contacted the Emergency Center of the Ministry of Health in Brazil. On January 27, 2020, Brazil implemented a nationwide contingency plan. On January 30, 2020, the WHO declared COVID-19 a Public Health Emergency of International Concern and, on February 3, 2020, Brazil also declared COVID-19 a Public Health Emergency of International Concern [1, 2].

The context in which COVID-19 appeared in Brazil includes infectious diseases, chronic conditions and risk factors (smoking, obesity, physical inactivity, etc.), and violence, among other causes related to sociocultural and environmental problems present in the country [2].

A study conducted with physicians and nurses in Hubei, China, using a phenomenological approach showed the physical suffering experienced by nurses due to the constant use of PPE, and excessive workload, often lead to reduced immunity and contamination with COVID-19 or other diseases. Predominance of fear was also highlighted as a constant aspect among healthcare providers, since previously unknown types of transmission caused this phenomenon, combined with the insecurity of all healthcare providers. In this Chinese study, the results showed three themes that were correlated with the theoretical framework used: being fully responsible for the wellbeing of patients (“this is my duty”), the challenges of working in COVID-19 wards, and resilience while facing challenges [3].

A study conducted in Canada and published in 2021 assessed the effect of the pandemic on the mental health of nurses of an intensive care unit (ICU) using semi-structured qualitative interviews. Inductive thematic analysis highlighted the predominance of anxiety, concern, anguish, and fear, which seemed to be related to rapid changes in policies and information; oppressive and unclear communication; meeting patient care needs in new ways while maintaining biosecurity; and managing personal and domestic commitments to oneself and family [4].

Frontline physicians fighting against the pandemic in ICUs experienced deep anguish and moral suffering as they were immersed in internal conflicts often going beyond the existential condition, with these phenomena triggered by the consequences of their decisions and having different causes for their occurrences. Diverging relations were also observed when experienced physicians or interns made decisions since the first group had more autonomy and control over physicians or interns made decisions since the first group had more autonomy and control over decisions and consequences [5]. Another study conducted in 13 Canadian universities assessed the perceptions and experiences of physicians while treating critically ill patients in the context of resource depletion during the first wave of the COVID-19 pandemic. The results highlighted the conditions that contributed to resource strain (e.g., continually worsening pandemic conditions); implications of resource strain on intensive care physicians personally (e.g., safety concerns) and professionally (e.g., practice change); and enablers of resource sufficiency [6].

Considering the COVID-19 pandemic, some studies in the scientific literature have addressed mental health issues among healthcare providers who treated patients diagnosed with COVID-19, with results showing different paths. One study presented a mix of negative emotions (fear, anxiety, concern) in the initial stages of the disease, as well as positive emotions (trust in medical and governmental capacity, trust in self-prevention, and control ability after training and practice) that emerged simultaneously during the experiences of providers involved with the clinical management of these patients [7].

According to a study conducted in Lebanon, healthcare providers became the most vulnerable population to contract the virus, representing more than 20% of people who contracted the disease.

Therefore, several emotions and fantasies related to the future were expressed by these professionals, including anxiety and concern, due to the risks they took, which exposed their family to possible infection [8]. Similarly, other challenges emerged due to the COVID-19 outbreak, causing many psychological and emotional issues and illnesses among healthcare providers treating patients with COVID-19 in China, the United States, Canada, Taiwan, and Hong Kong [9]. Many qualitative studies assessing this theme presented other relevant data about the most common consequences related to mental health during the quarantine period due to SARS-CoV-2, which had the predominance of post-traumatic stress disorder, depression, and abuse of alcohol and other substances [10]. Another important aspect was the misinformation and non-compliance that healthcare providers had to constantly deal with, whether in the medical context or at home. Since it was an unknown phenomenon, everyone had an opinion about it, without evidence of its impact and the possibility of controlling the disease and its advances in each patient [11].

A study conducted in Istanbul, Turkey, presented statements of nurses who, during the pandemic, withdrew from social life due to the risk of being stigmatized by society and due to the transmission of the disease. However, this isolation contributed to feelings of abandonment and loneliness, leading to more severe psychological distress because of a lack of psychosocial support.

Healthcare providers and their families felt stigmatized, irritated, stressed, afraid, guilty, helpless, lonely, tense, sad, and anxious during the quarantine and the pandemic process. They also presented avoidance behaviors [12].

Many COVID-19 patients showed feelings of gratitude during the pandemic. However, this feeling was expressed according to the clinical status of each patient. Overall, most of them reported negative emotions in the initial stage of the disease, gradually mixed with positive emotions, which constituted psychological adjustments experienced by these patients [13], a common element in this and other studies found in the literature.

The pandemic scenario in Brazil has particularities that need to be highlighted from a macropolitical perspective, as this scenario directly influences the results interpreted through the participants accounts in the present study. Brazil was considered an epicenter of the COVID-19 pandemic due to the worsening of the health situation caused by the politicization of the federal government [14]. Once again, the country was divided between those who were pro-vaccine and those who were anti-vaccine, and political issues stirred up in this context significantly influenced the decision-making of the general population, including healthcare teams. The robustness and advancement of a public health system, such as Brazil’s Sistema Único de Saúde (SUS), considered a model for emerging countries, created a fertile ground for various global inquiries when considering the dynamic democratic regime in contrast to the radical right-wing populism and its repercussions on the health and pandemic context [15, 16]. Although this topic is of notable relevance, this study does not focus on delving into political or sociological issues regarding politics and its macro impacts on public health in Brazil in the context of COVID-19.

Regarding the mental health of healthcare providers in Brazil during the pandemic, the PsicoVida project stood out. Its aim was to investigate the mental health of this group both cross-sectionally and longitudinally, with a particular focus on those working in hospitals and emergency units (Reference: https://www.uff.br/05-07-2021/cuidar-dos-que-cuidam-projeto-coordenado-pela-uff-investiga-saude- mental-de-profissionais-da-saude-durante-a-pandemia/.). Another study conducted with 151 frontline professionals indicated a 17.2% prevalence of PTSD. Previously diagnosed psychiatric disorders and the presence of PTSD showed a statistically significant association with higher scores of current depression, anxiety, and stress symptoms during the pandemic [17]. A cross-sectional study conducted in Brazil indicated a high prevalence of suicidal ideation, minor psychiatric disorders, and major depressive episodes among nursing professionals in the context of the pandemic [18]. The association with improved working conditions and biosafety measures were shown to be elements that could help manage this increase in mental illness in scenarios such as the COVID-19 pandemic [19].

The focus of the present study was, in general terms, to reveal the social, cultural, temporal, and political context in which the conflicting relationships and mechanisms of denial emerged, which we define ego defense or means of psychosocial adaptation.

## Method

The study design utilized the CQM, a conceptual approach to qualitative research originating from the Humanities. When applied in the health and educational areas, it is especially relevant for investigating the meanings that individuals assign to phenomena [20].

Unlike the natural sciences, which seek explanations and relationships of cause and effect regarding phenomena, the human sciences seek to understand the relationships of meaning that occur between phenomena [23]. Clinical-qualitative research, therefore aims to understand phenomena through the relationships of meaning that are established with them. A key characteristic is its specificity for the scope of settings that involve health care.

In this particular study, The CQM was applied to academic settings in the health sector. It enables the exploration and interpretation of emotional/psychological meanings that individuals (patients, family members, staff) attribute to phenomena experienced and observed in the process of illness, therapeutic care, and preventive care, as well as in the context of health education. In clinical-qualitative research, emphasis is placed on listening, observation, and both conscious and unconscious emotions, feelings, and anxieties, as well as the interaction with the researcher.

Establishing a minimal bond between the researcher and interviewee is essential to foster a quality interaction, enabling the interviewee to express themselves as spontaneously as possible within the framework and objective of the research.

In the CQM, senses and meanings are seen as the core of the study, that is, the core of the research lies in the researcher’s capture of meanings that the research subjects give to the phenomenon and in the interpretation that is made based on such elements. There is also the essential appreciation of anxieties, seen as fundamental, understanding anguish not only from a strictly clinical point of view but from an existentialist perspective, as “something that brings restlessness, which ends up limiting and restricting life” [20, 21]. The concerns and anxieties consciously or unconsciously expressed by the research subject, constitute highly relevant materials for understanding the meanings attributed to the phenomenon.

This was a humanistic study using the CQM [20], which enabled a better understanding of the meanings that intensive care physicians and nurses from a tertiary referral hospital at a public university in Southeastern Brazil attributed to their experiences while providing care to COVID-19 patients admitted to the ICU. A theoretical framework based on medical psychology was adopted for the analysis and discussion of findings. Since it was a Balintian data analysis, it was conducted from a psychodynamic perspective, which provides meaning to the statements.

The researcher, as a research instrument, offers their personality and internal availability to the research. More than that, they are implicated in it, because, as Bleger says, “when examining the lives of others, the review and examination of one’s own life, personality, conflicts and frustrations are directly implicated” [22]. Thus, bringing together data from the observational field, interview material, his notes and countertransference elements, he builds, like a bricoleur [22], understanding by using theoretical references and psychodynamic elements that can aid in the identification of meanings and the interviewees’ understandings about the phenomena. In psychodynamic studies, unconscious parts of the psyche are repositories of symbols, therefore, this process involves listening to the interviews at the time of data collection, focusing on the reports of the professionals’ experiences, and using a theoretical framework to support the interpretation of the psychodynamic aspects.

This research project involved members of the Laboratory of Clinical-Qualitative Research (LCQR) at the Universidade Estadual de Campinas, which includes a multidisciplinary team with physicians, nurses, psychologists, and dentists with experience in semi-structured interviews aimed at identifying core meanings in the floating readings of transcribed content. This article was developed according to the COREQ checklist criteria (consolidated criteria for reporting qualitative research). No software was used for data analysis.

Regarding the context in which the research was conducted, it was carried out in Brazil, which was considered an epicenter of the COVID-19 pandemic. As of August 12, 2021, the cumulative number of confirmed cases worldwide had surpassed 200 million, and the total number of deaths had exceeded 4.33 million. On the same date, the total number of COVID-19-related deaths in Brazil reached 560,000. Currently, the total number of COVID-19 deaths in Brazil stands at 712,889, according to data from the Ministry of Health. The total number of confirmed cases has reached 38,863,345. However, due to vaccination, the viral lethality rate is now 1.8%. The Brazilian government adopted a relatively relaxed mitigation strategy to handle the outbreak. To mitigate the impact on the economy and reduce the pressure on the healthcare system, Brazil implemented a more moderate approach to the outbreak [23, 24].

### Study location

This study focused on the COVID-19 ICU of the Hospital das Clínicas of the Universidade Estadual de Campinas, a university referral hospital that provides highly complex care to the general community nationwide, capturing real-time experiences of interviewed healthcare providers, as data collection occurred simultaneously with the COVID-19 pandemic. Although it is not an emergency service, continuity of care was identified. Therefore, a bond was created between physicians and nurses and their respective patients, given the unprecedented nature of the event, which helped facilitate the investigation of the emotional meanings in this care relationship between healthcare providers and COVID-19 patients. This hospital was chosen for this study because the researcher had previously conducted his master’s research in the same place.

The Universidade Estadual de Campinas (located in Campinas, a large inland city in the state of São Paulo) operates a referral hospital that treats more than 2 million people from neighboring areas and provides more than 60 university courses, including Nursing and Medicine.

### Sampling and recruiting

The study sample consisted of six interviewees: two physicians and four nurses. It employed purposive sampling and the inclusion criteria were: a physician or a nursing team professional, regardless of gender or age, providing care to intensive care unit patients with COVID-19 in the institution mentioned above; the interviewee needed to be introduced by the physician and nurse in charge of the respective care service to participate in the study; the interviewee had to agree with and sign the consent form after reading it in order to participate; the interviewee had to have proper physical, emotional, and intellectual conditions at the time of data collection, to ensure methodological validity, as expected when obtaining information from interviews.

The interviews were conducted only with participants who were referred by the department heads of the ICU, including the medical and nursing supervisors responsible for the COVID-19 ICU.

During the interviews, the researcher, drawing on their experience as a clinical psychologist, observed a logical structure in the interviewees’ speech. Subsequently, the interviews were subjected to content analysis by peers, who also confirmed that the interviewees maintained their physical, emotional, and intellectual functions intact, enabling their participation in the interviews.

Another point to consider is the fact that the study participants were active healthcare providers, which implies that the target audience would have physical, emotional, and intellectual conditions compatible with their professional duties and, consequently, preserved conditions to participate in the study through the interviews.

The researcher, who is the first author in the affiliation section, conducted all interviews remotely, complying with ethical guidelines. The researcher had visited the study site before the onset of the pandemic to learn more about the public university hospital prior to developing the research project and gaining familiarity with the study location. The researcher observed the work routine and took field notes, which subsequently supported the Clinical-Qualitative Content Analysis to understand the experiences reported by the interviewees. During this period of building relationships with the health teams and patients, the researcher established the first contact with the subject/environment, which helped them understand the investigated universe [15]. From the beginning of the data collection, in the second half of 2021, contact with the study participants (via email) was mediated by the heads of COVID-19 ICUs (a physician in charge of the medical team and a nurse in charge of the nursing team). The dates and times of the interviews were previously scheduled according to the preferences and convenience of the interviewees.

Sampling followed the criterion of theoretical saturation, which involved the inclusion of new participants until the researcher determined that collecting and analyzing additional data no longer provided new information about the study topic during interviews; i.e., the interviews began to reveal repeated patterns [25, 26].

### Data collection

The instruments used for data collection were semi-directed interviews with open-ended, in-depth questions and complementary field notes. The interview technique began with a triggering question: “I would like you to report your emotional experience during your work in the intensive care unit for patients with COVID-19.” During the interviews, follow-up questions were used to expand and clarify the statements, followed by finalizing questions (“Would you like to tell me anything else that we have not discussed? Would you like to ask any questions?”).

The data collection process (conducting online interviews) commenced on November 1, 2020 and concluded on May 31, 2021. Before starting each interview, the interviewer introduced themselves to the interviewee and read the entire consent form. Subsequently, they asked the interviewee if they had any questions and if they agreed to participate. Then the interviewee signed the form in duplicate and the interview began. The interviews had no pre-determined time limit. Every respondent participated in the study only once. These interviews were audio-recorded and later transcribed in full.

Due to the pandemic, the interviews were performed remotely. The physician and the nurse in charge of the COVID-19 ICU teams allowed the researcher to contact the study participants and send a study invitation by email; subsequently they provided their telephone numbers. Next, a text message was sent to selected participants explaining the study objectives and inviting them to an individual online interview via Google Meet. The email sent to participants included a link to access the online consent form, if they accepted the invitation. After agreeing to take part, the remaining interviews were scheduled and performed online on Google Meet platform. The full transcription was performed by a scholarship student, trained in the technique and theory described by the LCQR and consolidated in the scientific literature.

### Ethical approval

The project was approved by the Research Ethics Committee of the School of Medical Sciences, Universidade Estadual de Campinas, São Paulo and the Comissão Nacional de Ética em Pesquisa–Conep, under Number. 4,104,013 of June 22, 2020. All procedures complied with ethical standards. Data collection (conducting online interviews) commenced on November 1, 2020 and concluded on May 31, 2021.

### Data analysis

Regarding the specificity of the methodology used, since it is a highly interpretative method, the authors initially processed the data through floating reading (explained in greater detail below).

The analyses of the clinical-qualitative content were conducted during meetings with the research group. The contents that formed the core themes were highlighted, and the data and interpretations were subjected to peer validation, thereby confirming them as categories of analysis, which were presented as the results of the present study.

**Fig 1:**
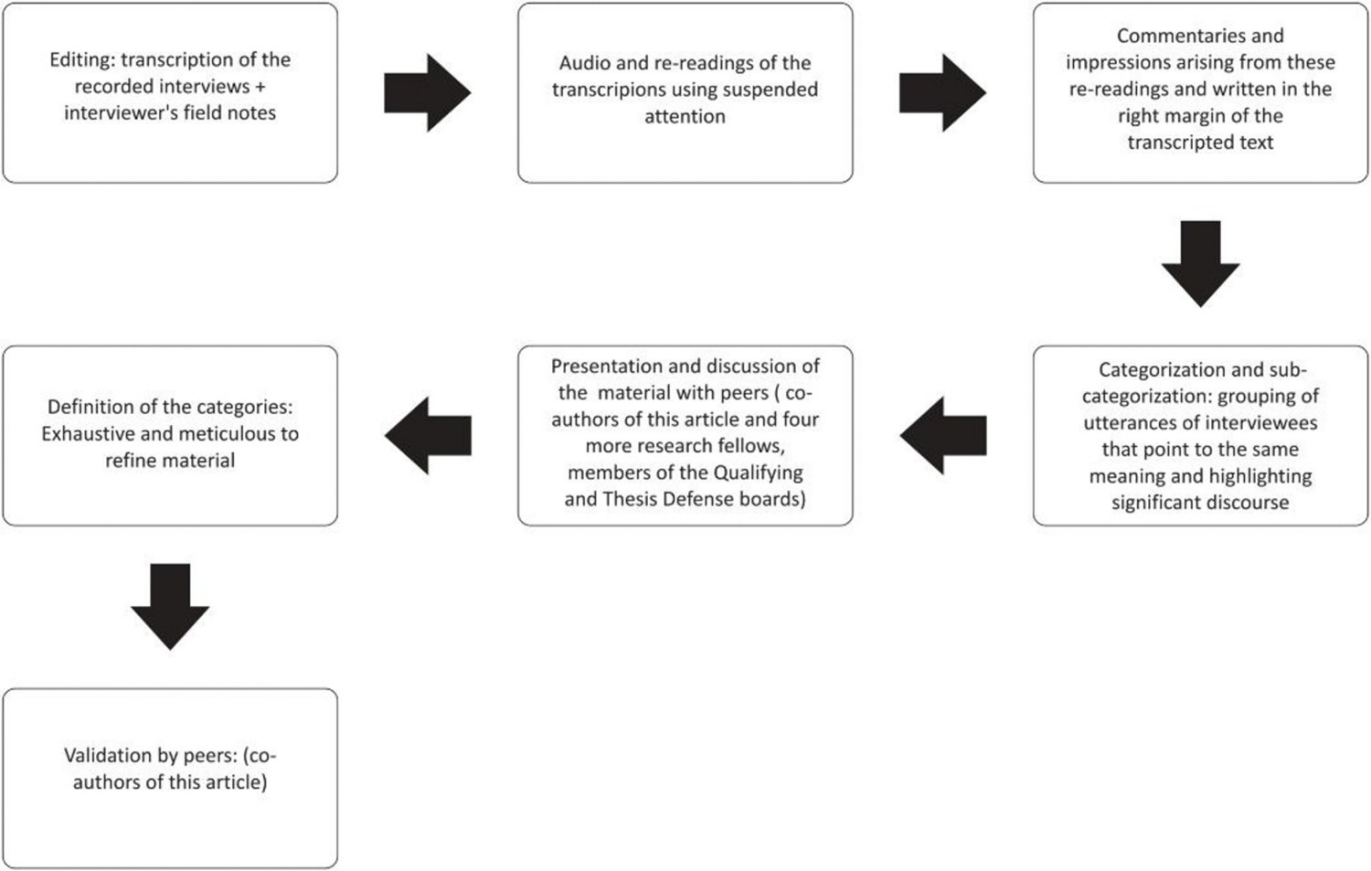
Flowchart of the Clinical-Qualitative Content Analysis.

Data were analyzed using Clinical-Qualitative Content Analysis. The corpus is the set of interviews transcribed in full and the additional notes made after the fieldwork. Using the Seven Steps technique by Faria-Schützer [27], the transcribed material was subjected to floating reading and re-reading when units of analysis (codes) emerged. These units were then consolidated into discussion categories that were validated by peers in meetings of the research group to which the authors are filiated. Fig 1 illustrates this analysis. All co-authors participated in the project, from Stage 2 until its completion. Data were validated by peer researchers who are members of the Laboratory of Clinical-Qualitative Research (LCQR).

The Clinical-Qualitative Content Analysis was conducted in seven steps. Initially, an inductive process was used, in which categories and explanatory hypotheses were formulated based on the data collected in relation to the research objectives. First, the interviews and the researcher’s observations from field notes were transcribed in full. Next, a floating reading of the material was performed, allowing for the observation of Freudian slips, metaphors, and symbolism in the interviewees’ accounts. The floating reading is the step in which the researcher engages in a relaxed reading of the participants’ accounts. It is a way of reading while "surfing," without prioritizing any specific elements of the speech: the researcher lets their unconscious activity flow freely, suspending motivations that might direct attention [28].

Each reading begins with an antidote to intrusive influences that could distort the analysis process: "No memory. No desire." The next step involved selecting meaning units guided by the questions the research aimed to answer. The following phase was the categorization process, where issues were identified according to their level of intimacy or proximity, organizing the themes that expressed important meanings and elaborations and that aligned with the study’s aim of producing new knowledge [27]. The validation of the findings was conducted by peers from the Laboratory of Clinical Qualitative Research at the Department of Medical Sciences of the University of Campinas and through national and international scientific events.

To interpret the collected data, the Balintian approach was used as a theoretical support framework, in conjunction with psychodynamics. In his 1957 work, "The Doctor, His Patient and the Illness" [29], the Hungarian physician and psychoanalyst proposed highly pertinent reflections from an emotional and psychological standpoint regarding doctors and their patients, their life stories, and how they and their colleagues were affected and influenced during the relationships established with patients, whether in clinics, hospitals, or daily life. Balint suggests a focus on the affective-symbolic dimension that develops between the doctor, the patient, and the illness process. He also presents concerns about medical education and clinical practice, which mobilize complex emotional structures, including ego defense mechanisms such as denial, transference, and countertransference, among others.

Balintian data analysis focuses on the emotional and psychological dimensions and management that emerge in the relationship between doctor/nurse and patient, particularly in the care interactions related to COVID-19 in the present study. Regarding the psychodynamic perspective, it involves an analytical view of the ego defense mechanisms that are unconsciously triggered an expressed in the participants’ speech excerpts. Additionally, a perspective related to coping strategies concerning the emotional impacts caused by the pandemic, as highlighted in the accounts, is used.

## Results

Six participants were interviewed. All healthcare providers accepted the invitation to take part in this study. The interviews were conducted at times chosen by the participants, outside of their working hours. The meetings were online using individual interviews. The interviews lasted 30 to 55 minutes. In CQR the researchers do not ask participants to make corrections to transcript reports, nor to provide feedback on the findings constructed by the researchers. The authors work with the discourse as it was spontaneously said. It is understood that the person’s truth emerges from free thoughts. Incorrect information does not exist. A lapsus linguae reveals more of the interviewees’ mental universe than a corrected and aseptic revision by them. The participants can be informed that feedback on the findings might occur by reading the results once published in the future. There was no need to repeat any interview.

Physicians and nurses were invited to speak freely about what they felt regarding their experiences and the implications of providing care to patients with COVID-19 in the ICU. Four categories emerged from data analysis: Category 1 –Psychic time and rudimentary symbolizations of personal experience during the pandemic; Category 2 –Denial as a psychological defense or psychosocial adaptation mechanism? Category 3 –Tension and family support: triggers of ambivalent emotional experiences; and 4: The feeling of insecurity: from technique to the affective dimension.

In order to maintain participant confidentiality, every interviewee was designated with the letter “E” plus a number corresponding to the order of the interviewees. Table 1 shows the biodemographic characteristics of the studied sample.

**Table 1:** Biodemographic characterization of the participants, Campinas, SP, 2023.

Participants	Sex	Age	Occupation	Years in this	Marital status
		(years)		occupation (years)	
E1	Female	50	Nurse	18	Married
E2	Female	45	Nurse	19	Married
E3	Male	44	Physician	20	Divorced
E4	Female	48	Nurse	10	Consensual union
E5	Male	33	Physician	4	Single
E6	Male	50	Nurse	30	Consensual union

### Psychic time and rudimentary symbolizations of personal experience during the pandemic

The statements of participants revealed some urgency to describe details of the experience of working in the health context during the COVID-19 pandemic and the negative impacts caused by the pandemic, both from work and personal perspectives. This urgency contributed to the use of lapses, metaphors, and simple rather than complex, symbols to help them express what was hidden in the semi-directed interview with open ended deep questions, information that is stored between the lines of the discourse by the study participants.

E1 –“*Emotionally speaking, I’ll tell you that it’s tough, I cried many times. Because we didn’t know… you know that uncertainty, especially when I got infected”.*

E1 –*“We were really afraid of dealing with patients with COVID*, *we were always dressed like we were going to space*, *or with this style*, *wearing a mask and everything*. *Over time*, *we were constantly afraid of getting close to the patient”*.

### Denial as a psychological defense or a psychosocial adaptation mechanism?

The statements reveal denial in moments of deep distress during the pandemic, highlighting the impact of experiencing such a reality that mobilized mechanisms at moments when the subject is affected by something they cannot face or does not know how to deal with.

Refusal to accept the invisible and unknown, potentially lethal, leads to disbelief; after all, refusal to believe or accept that something terrible is happening seems better (fancifully) than dealing rationally with the pandemic reality and its implications.

E6 –*“It’s getting closer and closer to us. Nowadays, we really believe it’s serious. At first, I didn’t truly believe it, I thought it was more of a media thing. Because, in the hospital, we didn’t have many people dying from COVID. Why? Because the treatment the facilities provides is the best in the region, it’s a referral hospital. But now I know how serious the situation is”.*

E6 –*“Now*, *in the second wave*, *I actually became aware of that”*.

### Tension and family support: Triggers of ambivalent emotional experiences

In the speech of E5, different meanings were identified regarding his relationships with patients in the ICU (seen and treated like family) and the absence of family members during the pandemic, mixed with the tension arising from self-care, care for others, and the fear of contaminating close family members.

The distress of healthcare providers about keeping their respective families healthy was also a strong issue. Fear and insecurity were prevalent. On the other hand, as the healthcare provider cares for and is close to the patient in the ICU, the patient is also close to the physician and nurse, consolidating and fulfilling mutual emotional needs due to the context of health and illness, and the absence of or impossibility of seeing their family members.

E5 –*“My feeling was already something like ‘I need support from my relatives,’ ‘I need to be supported,’ and in fact, my wife only offered fear”.*

E1 –*“When we hear about a colleague who died*, *who used to work with us*, *and we knew the person*, *it’s really sad*. *I thought it was the darkest phase of my professional life”*.

### The feeling of insecurity: From technique to affective dimension

The role of the collective ego is identified in the speech of E2 about how she felt safe when she entered the room of COVID-19 patients with other professionals from the multidisciplinary team.

Both E1 and E2 described technical relationships mediated by an affective dimension. This was collectivity versus individuality. The collective ego, i.e., the unconscious egoic experience of a group, is strengthened and consolidated by the cohesion between the members of the healthcare provider group.

E1 –*“The ICU is a place where a lot of people die, but nothing like what we’ve seen in this period.”*

E2 –*“I was afraid to work in the ICU and come back home with something*, *because I have children*, *my mother lives with me*, *she’s 71 years old*, *suffer from hypertension*, *I mean*, *she has underlying medical conditions that make her vulnerable to being contaminated*. *So I was afraid of contaminating someone*, *or contaminating myself”*.

*So*, *we started to care for our colleagues*, *you know*. *Or to check on our colleagues who were hospitalized elsewhere* (E1)”.

The technique referred to here as the title of the category pertains to the instrumental dimension, that is, the techniques that doctors and nurses use in the management of patients in ICUs.

With the advent of the pandemic, this dimension, previously more restricted to technical aspects, was transformed, and affective elements became part of the ICU context among healthcare providers and patients. This is evident in the excerpt from the account of E1 highlighted above.

## Discussion

Based on the categories of analysis presented, a distinction is drawn between the chronological time and the psychic time experienced by the study participants. Each has its own uniqueness in terms of how they processed such intense events, capable of mobilizing highly complex psychic and emotional structures, as was the case during the COVID-19 pandemic.

On one hand, we observe the denial of the pandemic’s severity, as reported by the interviewees. This denial, interpreted psychodynamically, is conceived and anchored in political discourses that, at that time, did not acknowledge the severity of the health crisis that Brazil and the world were facing. Conversely, as a way to continue performing their professional duties, the healthcare providers denied the reality as it was, as a means of making it possible to carry out their tasks without succumbing emotionally to the intense emotional burden and the mental health impacts caused by the deaths, losses, and restrictions imposed by the coronavirus.

Furthermore, another significant transformation occurred in the family relationships of healthcare providers during the pandemic. The duality of familial support and tension, or the ambivalence present in these relationships, points to a conflictive path from an emotional perspective.

Fear predominated, and with it, feelings of loss, restrictions, anxiety, and suffering led intensivist doctors and nurses to place expectations on their families that were not fulfilled, as their families were also experiencing the same feelings and anxieties.

The clinical care approach was also transformed. Prior to the pandemic, the interviewed professionals possessed adequate technical skills to perform their routine activities in the ICU, and that was enough. However, in the pandemic scenario, which was atypical, technical skills alone proved insufficient for the care of hospitalized patients. The pandemic situation itself demanded more, leading to the increased prominence of the affective dimension. That is, the dynamics of transference and countertransference between healthcare providers and patients became more frequent, transforming the clinical care dynamics.

In this study, we sought to provide an assessment with a qualitative design, an interpretation of the psychodynamic perspective involved in the results obtained. A duality stands out regarding the chronological time, respecting the order in which events occurred, and the psychic time, which is not linear and does not respect a logical sequence, but rather relies on affective disposition, activated defense mechanisms, and ego functions available at the time adverse phenomena occur.

The qualitative studies presented throughout this work, duly referenced at the end of the article, demonstrate the diversity of qualitative research on this topic. The participants of this study, due to the trauma experienced, presented a stronger need to speak rather than to listen. The interview was the first moment of a raw experience; refined after the participants started talking about it. The rawness of the pandemic experience gave rise to an eloquent and realistic discourse, reflecting the scarcity of symbols, at least until the interviews were carried out. In their statements, the interviewees described in detail their emotional experiences during the pandemic, highlighting significant milestones in their life stories and professional experiences.

From an interpretative analytical perspective, the testimonies carry meanings of experiences that are more subject to existential (phenomenological) inferences than psychoanalytic (revealing hidden symbolic elements within the discourse). Amid such intense emotional experiences, actions of defense and adaptive mechanisms of ego protection were observed.

Since the statements address dualities, we present the following questions: Would defense of the ego in the face of something unexpected that causes such pain and suffering and therefore becomes impossible to accept at that moment be denial, given the emotional and psychological conditions of the subject in question? Or would the possibility of adaptation, of existing and resisting in an unfavorable context involving losses, abrupt changes, and mourning be denial?

It is necessary to understand that, according to Laplanche [30], a defense mechanism consists of “different types of operations in which defense mechanisms can be specified. There is no disagreement that defense mechanisms are used by the ego, but the theoretical question remains open as to whether their use always presupposes the existence of an organized ego that supports them. The term mechanism has been used by Freud from the beginning to express the fact that psychic phenomena present articulations susceptible to scientific observation and analysis” [30].

In Freud’s work, Denial (*verleugnen* in German) designates “a process by which the subject, although formulating one of their until now repressed desires, thoughts, or feelings, continues to defend themself from it by denying that it belongs to them” [30]. We understand that this denial is expressed as a result of the disease presented by the external world during the COVID-19 pandemic, as mentioned by the doctors and nurses. Faced with this reality, the fear of the unknown and anxiety are inevitable and threaten the ego. Now, with the awareness of this, the split in the salutogenic clinical practice emerges, with the advent of the pandemic and its macro physical and emotional impact.

According to Balint [31], the “apostolic mission or function means that every physician has a vague but almost unshakable idea about how an ill patient should behave”; on the other hand, “a particularly important aspect of the apostolic function is the need that a physician feels to prove to the patient, to the whole world, and, above all, to himself, that he is a good doctor, a kind professional, worthy of trust, and able to help [31]”. We understand that, in the apostolic mission or function in a pandemic context, where the patient’s behavior is unexpected and unpredictable, the physician finds himself in internal conflict, with a threatened ego.

Frustration, powerlessness, and inability to heal, save, and protect the patient from the virus make the apostolic function impossible, giving way to denial, which becomes necessary and capable of momentarily replacing this function so as not to diminish the healing power. This power is incorporated into the healthcare provider who has always healed, but who could not do that during the pandemic. The demand is the denial of the present moment, waiting for something that will place him/her as a good doctor/nurse, a kind trustworthy professional that is able to help.

During the course of the pandemic, the interviewees had their lives naturally modified and their relationships redefined, enabling them to continue working in a context immersed in pain and loss. One of the interviewees lost members of his family and co-workers and, as a result, work relationships began to be humanized, so that both the medical and nursing teams started to look at and consider their peers as part of their families, as everyone spent most of their time in hospitals, rather than at home with their families.

“Compassion fatigue may be common among professionals working in disaster and pandemic care environments, with the frequent presence of human pain and suffering” [29, 32]. “It may be related to a general decrease in well-being, inability to cope with the exposed conditions, and intense absorption of patient suffering” [29, 32, 33].

The statements indicate that fear of contamination among the health team and their families led to compassion fatigue, creating a complex scenario that required everyone to utilize new strategies. These strategies caused vicarious trauma, with care designed for hospitalized patients and transference and countertransference relationships between healthcare providers and patients. In psychoanalysis, transference “designates the process through which unconscious desires are actualized about certain objects within the framework of a certain type of relationship established with them and, eminently, within the framework of the analytical relationship” [32]. And countertransference is “a set of unconscious reactions of the analyst to the analyzed person and, more particularly, to the transference of that person” [34].

Merriam-Webster [35, 36] defines vicarious as “performed or suffered by one person as a substitute for another or to the benefit or advantage of another.” Trauma [36, 37] is defined as “an abnormal psychological or behavioral state resulting from severe mental or emotional stress or injury caused by an extrinsic agent.” Therefore, we can conclude that, vicarious traumatization is a transformation in the self of a trauma worker or helper that results from an empathic engagement with traumatized clients and their accounts of traumatic experiences. It is a special form of countertransference, stimulated by the exposure to another person’s traumatic material.

The perception of collectivity was a care strategy for professionals who treated patients with COVID-19. The diversity of unexpected phenomena of the pandemic caused adverse relationships that contributed to tensions between feelings of individuality versus collectivity in the health context.

An individual who is part of a group “acquires, solely due to numerical conditions, a feeling of invincible power that allows him to surrender to instincts that, if he were alone, he would have compulsorily maintained under coercion” [38, 39].

In a group, an individual is placed under conditions that allow him to eliminate repressions from his unconscious instinctual impulses. Social anxiety constitutes the essence of what is called conscience [37]. Contagion is a phenomenon whose presence is easy to establish and difficult to explain” […]. In a group, every feeling and every act is contagious to such a degree that an individual readily sacrifices their personal interest for the collective interest [35].

The most notable peculiarity presented by a psychological group is the following: “whoever the individuals constituting it are, no matter how similar or dissimilar their way of life is, their occupations, their character or intelligence, the fact that they have become a group places them in possession of a kind of collective mind that makes them feel, think, and act in a very different way than each member, as taken individually, would feel, think, and act if they were in a state of isolation” [35].

With the advent of the pandemic, various changes and disruptions in clinical and care services have been observed. Numerous effective strategies for controlling and managing pandemics have been identified based on experience with COVID-19, such as professional knowledge of the health crisis to be faced and addressed [39]. Another strategy is the possibility of considering interventions focusing on demand (which arises at the peak of the crisis); organizational planning factors; and individual characteristics of frontline personnel (e.g., experience in other health crisis contexts) [40].

However, the findings of this study highlight a focus on the mental health of healthcare professionals in unexpected scenarios, demonstrating that the process of mental and emotional elaboration requires time and does not follow the logical and rational sequence of events. There is a need for a psychic reserve as a defense against the negative impacts that such scenarios, like the pandemic, cause. It becomes essential to seek psychological support and care in advance, through group meetings, such as Balint groups, which focus on discussing and understanding elements of an emotional and symbolic nature, rather than solely addressing the physical or pathological dimensions of the doctor/nurse and patient relationship.

Our conclusions indicate that the interviewees were driven by actions of denial and anger. According to Kübler-Ross [41], the denial phase shows a decrease in the relevance of libido in the event itself, even if momentarily; and revolt stood out due to anger and non-acceptance of the reality experienced, as they were being forced to live and work in the pandemic, taking risks while saving lives. Our findings suggest that the emotional meanings of their work experiences were identified in these two initial phases of representative reactions.

## Conclusions

The statements of participants showed a lack of symbolization. Based on prior qualitative studies, more descriptive analyses highlight scenarios where healthcare providers had significant harm, considering the psychological dimension regarding the COVID-19 pandemic. We highlight that psychic and cultural representation takes time to occur.

Furthermore, new knowledge obtained in our study has theoretical implications for teaching and/or reskilling activities, including practical applications of our findings to psychological management in clinical and group settings, assessment of emotional experiences in healthcare practice and institutional policies, as well as relevance of new research proposals based on our results.

### Strengths and limitations of the study

The greatest strength of this study, in terms of its choice of a consistent theme, lies in the fact that it arises from real demands in the empirical field. Clinical professionals, upon noticing the emergence of humanistic issues, and thus wishing to understand the mental universe of patients, family members or staff (themselves), ask us, qualitative researchers, to investigate the meanings of the speeches and postures of such people. Therefore, we assert that the focus of the object arises in clinical practice, and it is to the clinician that the discoveries must return. Another strong point of this manuscript is the mention of the working hypotheses, as it is this methodological step that guided the phases of the investigation. Unfortunately, original articles are published with hypotheses omitted, both in many qualitative studies and in some experimental or population studies.

Strengths of our study are: the research method, which is based on an interpretative and not descriptive analysis of the collected material; the study location in Southeastern Brazil, in a public referral hospital in a renowned university, which treats thousands of patients with different health needs; the historical moment in which the study was conducted, during the peak of the pandemic from 2020 to 2021; and the selected sample comprising intensive care physicians and nurses who described their emotional experiences and related meanings in the context of COVID-19.

The study limitations include: sampling challenges, since the invited professionals initially thought it was an epidemiological study; and given that it is a qualitative study, which analyzes a reality in depth, data are not statistical and are not generalizable.

## Supporting information

S1 Appendix (DOCX)
