# Supplementary material for: “We started to care for our colleagues”: A qualitative study of statements by physicians and nurses from a COVID-19 ICU of a public university hospital in the Southeast region of Brazil
Source: PLOS Ment Health. 2025 Feb 11;2(2):e0000248. doi: 10.1371/journal.pmen.0000248 (PMC12798264; doi:10.1371/journal.pmen.0000248)
Supplement: S1 Appendix — (DOCX) [file pmen.0000248.s001.docx]

**Appendix**

**Interview and Virtual Questionnaire Data (Main Instrument) (Semi-structured Interview with Open-ended Questions)**

1. Trigger Question: Please describe your emotional experience while providing intensive care and treatment to patients hospitalized with COVID-19.

2. What patient behaviors and attitudes most emotionally affect the team of professionals at your facility? Please comment.

3. How does this experience as a healthcare provider impact your values and worldview?

4. When you return home, and particularly at bedtime, what thoughts typically come to mind about your day at the hospital?

5. As a professional, how do you manage your emotions and overall mental health?

6. How do your family members and acquaintances perceive your work in this type of care?
